# Dietary Habit Changes in Preclinical Medical Students

**DOI:** 10.7759/cureus.97240

**Published:** 2025-11-19

**Authors:** Shravya Yarlagadda, Izabella Hilmi, Megan Mobley, Lauren Cobbs

**Affiliations:** 1 School of Medicine, Texas Tech University Health Sciences Center, Lubbock, USA

**Keywords:** dietary habits, graduate student diet, medical student dietary habits, medical student health, medical student nutrition

## Abstract

Background

Dietary habits are essential to health and significant contributors to multiple chronic health conditions, including diabetes and hypertension. Medical students represent a unique population that is learning the obstacles predisposing their future patients to poor dietary habits, while also facing many of the same obstacles themselves. This study focused on preclinical medical students’ dietary habits before and after starting medical school.

Methodology

In this study, data were collected through an online survey instrument sent to all medical students at the Texas Tech University Health Sciences Center.

Results

Responses revealed a decline in meal regularity, balanced meal structures, and food access since the start of medical school. Further, students reported time and cost as key obstacles hindering healthy dietary habits. Despite this decline in healthy dietary habits, our respondents believed that their dietary habits did not change for the worse. The lack of awareness of their dietary changes exemplifies the difficulty of assessing a healthy diet.

Conclusions

These results indicate places where medical schools can support their students, specifically by providing healthy lunches and snack options, and streamlining access to information about school and community food resources. Further, integrating educational initiatives encourages healthy eating habits, provides meal prep-friendly and affordable recipes, and connects students to existing financial resources. Facilitating healthy dietary practices in medical students not only aids this vulnerable population but also contributes to better dietary advice for the future patients these students will provide for.

## Introduction

As the medical school curriculum places increasing emphasis on the link between diet and chronic health conditions, including obesity and type 2 diabetes, medical students are positioned to counsel future patients on healthier lifestyle choices. However, despite this knowledge, one study suggests that medical students do not incorporate this information into their lives [1]. We further explored the dietary habits of preclinical medical students by assessing their habits before and after starting medical school.

Medical students face several obstacles hindering healthy dietary habits, notably demanding schedules, financial strain, and educational stressors [2]. These obstacles are listed as significant contributors to unhealthy dietary choices and habits [3]. Significant time burdens are associated with the increased consumption of fast food or packaged food items [4]. This results in decreased nutritional value and sometimes increased prices. For the already financially burdened medical student population, this trade is felt to be heavier. With nutrient-dense foods generally set at higher prices and associated with more kitchen preparation time, faster and cheaper packaged and fast food options quickly become easier choices [5]. These obstacles could predispose medical students to unhealthy eating habits, including skipping meals, excessive dependence on fast food, and time-saving packaged meals. Our study aimed to further investigate these issues, specifically focusing on how the transition into medical school influences meal choices, structure, and content. By examining these obstacles, we sought to provide a deeper understanding of the dietary struggles faced by medical students in their preclinical years.

## Materials and methods

Survey

An online survey named “TTUHSC School of Medicine Patients, Physicians & Populations (P3) 1 Honors Project Omnibus Survey 2024” was sent to the Texas Tech University Health Sciences Center (TTUHSC) School of Medicine. The overall Omnibus survey included 21 sets of roughly 10 questions and received a total of 423 responses. This survey was one of the 21 sets, and the questions used for this study received 166 responses. All students received an email containing a link to the survey. Participation was optional, and no incentives were offered to complete the survey. This project was approved for exempt review by the TTUHSC Institutional Review Board (IRB#: IRB-FY2024-136).

Population of interest

The population of interest was preclinical medical students. The differences in scheduling and environment between preclinical and clinical medical students prompted a focus on one group. Furthermore, preclinical students represent a population adapting to the changes that come with medical school, and the dietary habits that changed at this stage were the target of this project.

Questions

The question set consisted of nine questions in total (Table 1). The first two questions addressed students’ self-perceived dietary habits before starting medical school and during medical school. The first question contained five statements (Table 1) regarding eating habits before medical school. In the second question, the same statements were provided regarding eating habits since the start of medical school.

**Table 1:** Omnibus survey questions with associated possible answer choices and format.

Question	Answer type and choices/statements
Please consider the following descriptions of eating habits. Reflect on how much each describes you before starting medical school. Use a scale of 1 to 5, where 1 = Does not describe me at all and 5 = Describes me very well	A Likert scale from 1 to 5 for each statement. I regularly ate three meals daily. I ate something between my major meals of the day. The majority of my daily meals followed the balanced plate structure shown above. I engaged in behaviors associated with disordered eating (e.g., restrictive eating, binge eating, purging by vomiting or laxative misuse, compulsive exercise, etc.). My eating habits were influenced by barriers to accessing food (e.g., time, cost, transportation, dietary restrictions)
Please consider the following description of eating habits. Reflect on how much each describes you currently, since starting medical school. Use a scale of 1 to 5, where 1 = Does not describe me at all and 5 = Describes me very well	A Likert scale from 1 to 5 for each statement. I regularly eat three meals daily. I eat something between my major meals of the day. The majority of my daily meals follow the balanced plate structure shown above. I engage in behaviors associated with disordered eating (e.g., restrictive eating, binge eating, purging by vomiting or laxative misuse, compulsive exercise, etc.). My eating habits are influenced by barriers to accessing food (e.g., time, cost, transportation, dietary restrictions)
Indicate your level of agreement with the following statements about the food you typically eat now. Use a scale of 1 to 5, where 1 = Strongly disagree and 5 = Strongly agree	A Likert scale from 1 to 5 for each statement. The majority of my meals are home-cooked. The majority of my meals are fast food or take-out. (i.e., McDonald’s, Panda Express, Taco Bell, etc.). The majority of my meals are frozen or pre-packaged. The majority of my meals are from the University cafeteria. I regularly eat meals prepared by a meal delivery service (i.e., Hello Fresh, Blue Apron, Factor, Tempo, Every Plate, etc.)
Indicate your level of agreement with the following statements about the food you typically eat now. Use a scale of 1 to 5, where 1 = Strongly disagree and 5 = Strongly agree	A Likert scale from 1 to 5. Since starting medical school, my eating habits have changed for the worse
Do you follow any of the specific diets listed below (list all that apply)	Select all answers that apply. Vegan or Vegetarian, Kosher, Gluten-free, Halal, Other: fill in the blank
Which of the following presents the greatest barrier to your meeting a goal of healthy eating?	Select all answers that apply. Time, Cost, Transportation, Knowledge, None of these/No barriers
About how much do you spend per month on food for yourself?	Select one answer. Less than $100, $100–200, $200–300, $300–400, $400+, I don’t know my monthly food expenditure
How do you pay for the majority of your food?	Select all answers that apply. My parents/spouse support me. Personal funds (i.e., past savings, part-time job). Student loans. Food assistance program (i.e., SNAP, EBT, TTUHSC food pantry, governmental assistance program)
If you have experienced any barriers to healthy eating, have you accessed any of the resources listed below? Check all that apply.	Select all answers that apply. Not applicable to me. Applicable but have not tried. SNAP/EBT TTUHSC Helping Hands food pantry. Local community food pantries or food banks (i.e., South Plains Food Bank). Religious organization program. Food provided by TTUHSC organ

The students assessed these statements using a Likert scale. Participants could reply with numbers 1-5, with 1 indicating that the statement does not describe them at all and 5 indicating that it describes them very well. Transitioning into a new environment can alter many habits that people previously had. These five statements addressed only a few possible areas where dietary habits could change. Students may not regularly eat meals, or may frequently snack between or instead of eating meals, making balanced nutrition more challenging [6,7]. Medical students can face increased stress and negative emotions, and such psychological distress has been positively associated with disordered eating attitudes [8]. Finally, as students transition to a new place, their access to food may change depending on how similar their previous environment and resources are to now.

The next question addressed the types of food that preclinical students eat. The students were presented with statements covering different types (Table 1). Knowing where students get the majority of their food from allows insight into healthy eating choices and the time students feel they can put toward making food. Home-cooked meals are generally considered healthier than meals made outside the home, with frequent consumption of meals prepared away from home being associated with an increased risk of all-cause mortality [9]. However, students face many struggles with home cooking, despite its benefits, specifically, a lack of time, money, or cooking skills [10].

The final question using a Likert scale assessed the participants’ views on the change in their eating habits in medical school. Participants were presented with the prompt, “Since starting medical school, my eating habits have changed for the worse.” They could respond with a number from 1 to 5, with 1 indicating strongly disagree and 5 indicating strongly agree. This question provided a comparison of students’ perceptions of their habit changes and the results. A negative change was specifically asked about due to past research showing negative eating habits in other medical student populations. These include eating irregular meals, not eating enough fruits and vegetables, and eating unhealthy foods [7,11].

Question five looked at the types of diets that the participants adhered to. This was a select-all-that-apply question with possible answer choices of vegan or vegetarian, gluten-free, kosher, halal, or other, where participants could fill in a blank. Various diets can affect a person’s health and access to food. Plant-based diets can be beneficial or harmful. A healthy plant-based diet with lower amounts of animal foods, sugary drinks, snacks and desserts, refined grains, potatoes, and fruit juices is associated with lower risks of cardiovascular disease, cancer, and total mortality, whereas a plant-based diet that does not follow these characteristics is associated with a higher risk [12]. A gluten-free diet is important for those who medically need to follow one and can improve or treat diseases, such as Celiac disease and gluten ataxia [13]. However, it comes with challenges, such as nutritional deficiencies and high costs [13]. Kosher and halal diets have challenges with the availability of foods that follow their religious guidelines. Many Muslims struggle to access halal food, especially if they are already food insecure, which affects their ability to have proper nutrition [14]. Jewish people who follow kosher guidelines can have similar issues with finding food because of the cost of kosher meals and ingredients [15].

Question six addressed whether the students had barriers to eating a healthy diet. This was a select-all-that-apply question with possible answer choices for time, cost, transportation, knowledge, and none of the barriers. Barriers to accessing high-quality food can affect health and physical and mental performance. Students with trouble accessing food can have physical and mental health problems, lower academic performance, and may not complete their academic studies [16].

The next question assessed how much students spent on food per month. Participants could select one of these answer choices: less than $100, $100-200, $200-300, $300-400, $400+, and I don’t know my monthly food expenditure. Increasing food prices can make it difficult for people to afford high-quality and healthy food. Higher prices can increase the risk of undernutrition, which falls under the category of malnutrition [17]. In other medical school institutions, malnutrition was found to have a prevalence of 29.5%, which shows the importance of evaluating medical students for this condition [18].

The second-to-last question examined how participants paid for their food. Participants could select one of the following choices: my parents/spouse supported me, personal funds, student loans, or food assistance programs. Students use various ways to afford food, and each can affect how much they spend on their food. Many students receive financial support from their families and financial aid, and many of them can work while in school [16]. Fewer students utilize food assistance programs to access and buy food [16].

The final question addressed whether students with barriers to accessing a healthy diet use resources to help gain access. The question was select-all-that-apply, and the available answers were not applicable to me, applicable but have not tried, SNAP/EBT, TTUHSC Helping Hands food pantry, local community food pantries or food banks, religious organization programs, food provided by TTUHSC organizations or electives, and others where participants could fill in the blank. Resources to access food can help people meet their nutritional needs. However, many people may not use the available resources. A previous study found that of the students they surveyed, only 11% of food-insecure students and 3% of at-risk students participated in food assistance programs [16]. This could be due to the need for knowledge of how to use these programs.

## Results

The results of the survey, as analyzed using a paired t-test comparing the students’ answers to each Likert scale question before starting medical school to the same students’ answers to those questions in the context of “since starting medical school,” illuminated some significant changes in the students’ eating habits.

The first Likert statement, “I regularly ate three meals daily,” was used to assess changes in eating frequency and adherence to the existing standard of eating three “square” meals a day (Table 2). The mean response before starting medical school was 3.69, while the mean response since starting medical school was 3.35, indicating a change of -0.34. This suggests that fewer people were eating the recommended three meals a day since starting medical school than those who did before medical school. The paired t-test produced a p-value of 0.000017, which is less than the significance value of 0.05, leading us to reject the null hypothesis that there was no change in favor of the alternative that there was a statistically significant negative change in the number of people who ate three meals a day.

**Table 2:** Survey responses to Likert-scale questions with associated means and paired t-test results.

Question	Before medical school	During medical school	Change	t-test
“I regularly ate 3 meals”	3.6867	3.3494	0.3373	0.00001738
“I ate something between my major meals of the day”	3.3454	3.1575	0.1878	0.0135
“The majority of my daily meals followed the USDA balanced plate structure”	3.0421	2.8614	0.1807	0.01198
“I engaged in behaviors associated with disordered eating (e.g., restrictive eating, binge eating, purging by vomiting or laxative misuse, compulsive exercise, etc.)”	1.6445	1.6325	0.012	0.8216
“My eating habits were influenced by barriers to accessing food (e.g., time, cost, transportation, dietary restriction)”	2.0843	2.5120	-0.4277	0

The second Likert statement, “I ate something between my major meals of the day,” was provided to account for those who have greater nutritional meals than the baseline of three meals per day, as well as to assess snacking behaviors (Table 2). The mean response before starting medical school was 3.35, while the mean response since starting medical school was 3.15, indicating a change of 0.187. This indicates that fewer people were eating in between major meals since starting medical school than those who did before medical school. The paired t-test produced a p-value of 0.014, which is less than the significance value of 0.05, leading us to reject the null hypothesis that there was no change in favor of the alternative that there was, in fact, a statistically significant change in the number of people who ate in between meals.

The third Likert-scale statement, “The majority of my daily meals followed the balanced plate structure shown,” assessed the quality and potential nutritional value of meals eaten by medical students before and after starting medical school (Table 2). The USDA “Balanced Plate” was provided with this statement as a template for what nutritionally balanced meals consist of to standardize the results and account for differences in each participant’s definition of “balanced” eating. The mean response before starting medical school was 3.04, while the mean response since starting medical school was 2.86, indicating a change of 0.180. This indicates that fewer people were eating balanced meals that followed the USDA standard since starting medical school than before medical school. The paired t-test produced a p-value of 0.012, which is less than the significance value of 0.05, leading us to reject the null hypothesis that there was no change in favor of the alternative, i.e., there was a statistically significant change in the number of people who ate balanced meals in medical school.

The fourth statement assessed using the Likert scale, “I engaged in behaviors associated with disordered eating (e.g., restrictive eating, binge eating, purging by vomiting or laxative misuse, compulsive exercise, etc.),” was used to assess the potential prevalence of disordered eating and the association between starting medical school and the adoption of disordered eating behaviors (Table 2). The mean response before starting medical school was 2.10, while the mean response since starting medical school was 1.63, indicating a change of 0.47. This indicated that fewer people engaged in behaviors associated with disordered eating since starting medical school than before medical school. The paired t-test produced a p-value of 0.82, which is greater than the significance value of 0.05, leading us to fail to reject the null hypothesis. This means that these results were not statistically significant and that there was a high probability that the resulting change was due to chance.

The fifth and final statement assessed using the Likert scale, “My eating habits were influenced by barriers to accessing food (e.g., time, cost, transportation, dietary restriction),” was used to assess the presence of barriers to accessing food (Table 2). The mean response before starting medical school was 2.08, while the mean response since starting medical school was 2.51, indicating a change of 0.43. This indicates that more people agreed that their eating habits were influenced by barriers since starting medical school than before starting medical school. The paired t-test produced a p-value of 0.0000013, which is less than the significance value of 0.05, leading us to reject the null hypothesis that there was no change in favor of the alternative that there was a statistically significant change in the number of people who felt as though there were barriers to accessing food.

Despite multiple statistically significant results indicating a decrease in the quality of eating habits in students since starting medical school, when presented with the final statement, “Since starting medical school, my eating habits have changed for the worse,” the participants disagreed (Table 3). The mean answer value was 2.3, which indicates more disagreement than agreement.

**Table 3:** Survey response to overall dietary habit change perception question with Likert-scale response average, standard deviation, and paired t-test result.

Answers	Average	SD	t-test
“Since starting Medical School my eating habits have changed for the worse”	2.297	1.3262	0.9754

The multiple-choice questions were designed to account for confounding variables that could be affecting eating habits and identify possible points of intervention. Among the participants, five followed a gluten-free diet, 15 followed a vegan or vegetarian diet, eight followed a halal diet, and one followed a kosher diet. Additionally, one participant responded using the “other” option that they do not eat pork (Table 4). When asked what the greatest barrier was to meeting goals of eating healthy, 105 (63.25%) individuals identified time as their greatest barrier, followed by cost, with 38 (22.89%) individuals. The final two options were knowledge (nine respondents) and no barriers (14 respondents) (Table 5). Regarding food expenditures, the majority of students reported spending $100-300 per month on food (Table 6). Most students reported that their parents or spouse supported them (48%) or that they paid with student loans (38%), while 14% responded that they used personal funds such as savings or a part-time job (Table 7). Finally, when asked whether they had used resources to overcome cost as a barrier, 15% of students stated that food provided by TTUHSC and associated organizations was significant to them, 3% accessed the TTUHSC Helping Hands food pantry, 3% used SNAP/EBT, and 7% stated that while they identified cost as a barrier, they did not know of or had not tried to access available resources (Table 8).

**Table 4:** Survey responses to the multiple-choice question “Do you follow any of the specific diets listed below (list all that apply)” and associated counts and percentages.

Answers for “Do you follow any of the specific diets listed below (list all that apply)”	Number of responses	Percentage
Gluten-free	5	0.0303
Vegan or vegetarian	15	0.0909
Halal	8	0.0485
Kosher	1	0.0061
None	136	0.8242
Total	165	-

**Table 5:** Survey responses to multiple choice question “Which of the following presents the greatest barrier to your meeting a goal of healthy eating?” and associated response counts and percentages.

Answers for “Which of the following presents the greatest barrier to your meeting a goal of healthy eating?”	Number of responses	Percentage
Time	105	0.6364
Knowledge	9	0.0545
Cost	38	0.2303
None of these/No barriers	14	0.0848
Total	165	-

**Table 6:** Survey responses to multiple choice question “About how much do you spend per month on food for yourself?” and associated response counts and percentages.

Answers for “About how much do you spend per month on food for yourself?”	Number of responses	Percentage
$100–200	54	0.3273
$200–300	54	0.3273
$300–400	28	0.1697
400+	11	0.0667
I do not know	18	0.1091
Total	165	-

**Table 7:** Responses to “How do you pay for the majority of your food?” and associated response counts and percentages.

Answers for “How do you pay for the majority of your food?”	Number of responses	Percentage
Student loans	63	0.3818
Personal funds (e.g., savings, part-time job)	23	0.1394
My parents/spouse support me	79	0.4788
Total	165	-

**Table 8:** Responses to the multiple-choice question “If you have experienced any barriers to healthy eating, have you accessed any of the resources listed below? Check all that apply” and associated response counts and percentages.

Answers for “If you have experienced any barriers to healthy eating, have you accessed any of the resources listed below? Check all that apply”	Number of responses	Percentage
SNAP/EBT	5	0.0303
Food provided by TTUHSC organizations or electives	25	0.1515
Applicable but have not tried	12	0.0727
Not applicable to me	118	0.7152
TTUHSC Helping Hands food pantry	5	0.0303
Total	165	-

## Discussion

It is important to note that there was no way to indicate whether those who did not agree with the first statement about eating three meals a day ate more than or fewer than three meals per day. Due to the nature of the survey, leading questions such as “I eat less than three meals a day” were excluded, as the study intended to assess decreased eating and skipping meals upon starting medical school. It is also important to acknowledge that the standard of eating three meals per day is a result of agrarian cultural factors, and that nutritional recommendations for the number of meals vary from person to person based on individual needs [19]. In this study, meals per day were used as a baseline because they provide a widely accepted standard that could be understood by all participants.

The changes observed in snacking behaviors are more difficult to categorize as “good” or “bad.” This study did not assess the nature or quality of foods eaten between meals, which is influenced by many individual, social, and environmental factors. Literature suggests that most snack foods are “energy-dense and nutrient-poor,” but there is also evidence that an emphasis on “healthful” eating can lead individuals to reach for nutrient-rich snacks. The relationship between snack frequency, nutritional value, satiety, overconsumption, and obesity is complex [20]. In addition, meal frequency and timing influence the synchronization of metabolic clocks, disruption of which is associated with increased risk of metabolic and cardiovascular disease. This study also suggests that reducing meal and snack frequency may positively affect the gut microbiome, which plays a role in systemic inflammation [19].

The finding that students ate fewer balanced meals is concerning, given the importance of the USDA “Balanced Plate” as a standard of healthy eating. Combined with the reported increase in barriers to food access, particularly time and cost, these results suggest that medical students are at risk for decreased diet quality after starting school. Interestingly, however, when asked directly whether their eating habits had worsened since starting medical school, students disagreed. This discrepancy may reflect a lack of awareness of gradual changes or the prevalence of imposter syndrome among medical students. Imposter syndrome is often associated with a desire to be perceived as doing well, which may lead students to deny worsening habits, even when objective measures suggest otherwise.

The multiple-choice questions confirmed that time and cost were the most significant barriers to healthy eating. Given that 63.25% of respondents identified time as their primary challenge, interventions aimed at time management, meal-prepping strategies, or providing healthier quick options may be most impactful. Cost was the second most frequently identified barrier, and most students reported spending $100-300 per month on food, often supported by loans or family members. This suggests that interventions should also emphasize cost-effective options within that budget range. The finding that many students relied on institutional food options such as cafeteria or vending machine offerings is particularly notable, as these meals are often calorie-dense, inexpensive, and nutritionally poor, which may contribute to unhealthy eating patterns. Expanding healthier options in these settings could therefore be an important intervention.

Finally, while relatively few students reported following restrictive diets such as gluten-free, vegan, halal, or kosher, acknowledging these subgroups is essential to ensuring that any proposed interventions are inclusive. Improved communication about existing resources, along with tailoring interventions to the time and cost challenges identified in this study, would likely have the greatest impact on improving medical students’ nutritional habits.

These results are relevant as they raise concerns about the ability of medical students to maintain healthy dietary behaviors throughout their education. Addressing these dietary challenges through targeted interventions, such as time management education, affordable meal preparation resources, and enhanced access to institutional food assistance programs, can help mitigate the obstacles faced by medical students and support their overall well-being. However, the results of this study are limited due to being collected from a single institution. Further studies that include more universities need to be conducted to assess trends in the medical student population.

## Conclusions

By fostering healthier habits among medical students, institutions can enhance their capacity to model and advocate for nutritional health, which could contribute to improved health literacy and dietary education for patients. Future research should explore the long-term implications of these findings and assess the efficacy of the implemented interventions.
